# Application of failure mode and effects analysis to treatment planning in scanned proton beam radiotherapy

**DOI:** 10.1186/1748-717X-8-127

**Published:** 2013-05-24

**Authors:** Marie Claire Cantone, Mario Ciocca, Francesco Dionisi, Piero Fossati, Stefano Lorentini, Marco Krengli, Silvia Molinelli, Roberto Orecchia, Marco Schwarz, Ivan Veronese, Viviana Vitolo

**Affiliations:** 1 Dipartimento di Fisica, Università degli Studi di Milano, Via Celoria 16, 20133, Milano, Italy; 2 Centro Nazionale di Adroterapia Oncologica (CNAO Foundation), via Campeggi 53, 27100, Pavia, Italy; 3 Agenzia Provinciale per la Protonterapia, Trento, Italy; 4 Department of Translational Medicine, University of Piemonte Orientale, Novara, Italy; 5 European Institute of Oncology, Università degli Studi di Milano, Milano, Italy; 6 Azienda Provinciale per i Servizi Sanitari, Trento, Italy

**Keywords:** Risk assessment, Patient safety, Accidental exposures, FMEA, Protons, Treatment planning

## Abstract

**Background:**

A multidisciplinary and multi-institutional working group applied the Failure Mode and Effects Analysis (FMEA) approach to the actively scanned proton beam radiotherapy process implemented at CNAO (Centro Nazionale di Adroterapia Oncologica), aiming at preventing accidental exposures to the patient.

**Methods:**

FMEA was applied to the treatment planning stage and consisted of three steps: i) identification of the involved sub-processes; ii) identification and ranking of the potential failure modes, together with their causes and effects, using the risk probability number (RPN) scoring system, iii) identification of additional safety measures to be proposed for process quality and safety improvement. RPN upper threshold for little concern of risk was set at 125.

**Results:**

Thirty-four sub-processes were identified, twenty-two of them were judged to be potentially prone to one or more failure modes. A total of forty-four failure modes were recognized, 52% of them characterized by an RPN score equal to 80 or higher. The threshold of 125 for RPN was exceeded in five cases only. The most critical sub-process appeared related to the delineation and correction of artefacts in planning CT data. Failures associated to that sub-process were inaccurate delineation of the artefacts and incorrect proton stopping power assignment to body regions. Other significant failure modes consisted of an outdated representation of the patient anatomy, an improper selection of beam direction and of the physical beam model or dose calculation grid. The main effects of these failures were represented by wrong dose distribution (i.e. deviating from the planned one) delivered to the patient. Additional strategies for risk mitigation, easily and immediately applicable, consisted of a systematic information collection about any known implanted prosthesis directly from each patient and enforcing a short interval time between CT scan and treatment start. Moreover, (i) the investigation of dedicated CT image reconstruction algorithms, (ii) further evaluation of treatment plan robustness and (iii) implementation of independent methods for dose calculation (such as Monte Carlo simulations) may represent novel solutions to increase patient safety.

**Conclusions:**

FMEA is a useful tool for prospective evaluation of patient safety in proton beam radiotherapy. The application of this method to the treatment planning stage lead to identify strategies for risk mitigation in addition to the safety measures already adopted in clinical practice.

## Background

New technologies have been introduced in radiation therapy (RT) with the aim of improving treatment outcome by means of dose distributions which conform more closely to the target volumes. Highly conformal dose distributions allow for dose escalation in the target volumes without increasing the dose to neighbouring normal tissues, or for a reduction in the dose to normal tissues without decreasing the dose to the target. In particular, actively scanned proton beams represent a novel irradiation technique taking full advantage from the physical interaction properties of these particles with tissues and advanced delivery modality to generate very sharp dose gradients in three dimensions, with many degrees of freedom available at the planning level.

The increased complexity related to the technological and process changes places new demands on quality assurance (QA) programs, as well as innovative instrumentation and detectors for beam characterization and checks [1-5]. Moreover, new approaches of safety culture are required, since complexity may also increase the sensitivity to uncertainties and risk for accidental exposures.

Radiotherapy-related errors are unfortunately not uncommon, even in the countries with the highest level of health-care resources [6]. A number of accidents in conventional external radiotherapy have been extensively investigated and the lessons learned have been disseminated by the International Atomic Energy Agency (IAEA), as well as the International Commission on Radiological Protection (ICRP) [7,8]. In order to fully assess and manage the risks of accidental exposures deriving from the use of innovative radiotherapy methodologies, retrospective approaches are not fully adequate, since they have the intrinsic limitation of being confined to the reported experiences, thus leaving unreported events or latent risks unaddressed. This is particularly true for new methodologies, for which safety reports may not be available. Prospective approaches, widely applied in high-risk industry, have to be implemented to find out all the elements that could go wrong and identify, *a priori*, all the potential hazards that might occur during a radiotherapy treatment. Recently, the interest in using these methodologies for safety assessment in complex medical practices, like modern radiotherapy, is gaining importance and the literature on this topic is rapidly increasing [9-13].

Following the general guidelines proposed by the World Health Organization (WHO) [6], the RT treatment process can be divided into ten stages: 1) assessment of patient, 2) decision to treat, 3) treatment protocol prescription, 4) positioning and immobilization, 5) simulation, imaging and volume determination, 6) planning, 7) treatment information transfer, 8) patient set-up, 9) treatment delivery, 10) treatment verification and monitoring.

The aim of this work was the application of the Failure Mode and Effects Analysis (FMEA) prospective approach to actively scanned proton beam radiotherapy, representing the most advanced irradiation modality using this type of particle. The specific processes implemented at CNAO Foundation (Centro Nazionale di Adroterapia Oncologica) was considered for the analysis. The study was focused on the treatment planning stage, since it was considered one of the most critical phases within the whole RT process, as also reported in the WHO Technical Manual [6]. The applied procedure included the definition of the involved sub-processes and fault trees, the assignment of a score for each potential failure mode and finally the suggestion of additional safety measures for process improvement. Risk analysis for the remaining nine stages, as well as for the commissioning of the treatment machine and treatment planning system (TPS), is out of the scope of this work and deserves being addressed elsewhere.

## Methods

### Failure modes and effects analysis (FMEA)

FMEA is a proactive risk analysis method, widely employed in industry and recently also recommended by the ICRP and Task Group 100 of the American Association of Physicists in Medicine (AAPM) as a powerful tool in modern radiation oncology [14].

In this study, FMEA was applied as a first step to identify all the sub-processes involved in the treatment planning stage of the proton beam RT process (i.e. the process tree), what could go wrong (i.e. the failure modes) and the potential causes and effects of each failure. Then, since the goal of FMEA is to rank the failure modes in order of importance, three indexes were assigned for each failure mode: the occurrence rating (O), the severity rating (S), and the detectability rating (D). The strategies and solutions currently applied at CNAO to mitigate the risk in the routine clinical practice are reported and were taken into account in the assessment of those indexes. A ten-point scale was used to score each category, ten being the number indicating the most severe, most frequent and least detectable failure mode, respectively. In particular, as a guideline, the ranking scales reported by Ford *et al.*[9] and already tested by some of the authors in a previous work were adopted [11]. Finally, the risk probability number (RPN) was calculated as the product of O, S and D attributes; for the failure modes showing higher RPN, thus indicating the areas of greatest concern in terms of potential risk, additional safety measures aiming at risk mitigation and process improvement were investigated. As for industrial applications and already applied in previous FMEA studies in RT [11,14], the value of 125 was considered as an RPN threshold below which the risk can be considered acceptable. However, it must be pointed out that this value, derived form industry, still remains somehow arbitrary when applied to RT and deserves further investigation.

The analysis was carried out by a multidisciplinary and multi-institutional team composed by experts in medical physics, radiation oncology, radiation dosimetry and protection, risk management. The operative methodology included a preparatory work mainly consisting of brainstorming in small groups and e-mail exchange, followed by several plenary meetings organized to delineate and discuss the process tree of the treatment planning stage, as well as identify the potential failure modes, causes, effects and conclusive additional safety measures. The risk indexes associated to each failure mode were initially conceived by members of the working group on an individual and independent basis (i.e. in “blind” mode), then collectively revised during a dedicated plenary session to reach general consensus.

### The treatment planning stage within the proton beam RT process

The RT process actually implemented at CNAO, hereafter briefly described, was assumed as a reference for the detailed definition of the process tree and the estimation of RPN numbers. CNAO is an Italian hospital-based facility using a synchrotron to accelerate proton and carbon ion beams [15-17]. Spill-to-spill capability of beam energy variation, as well as pencil beam scanning in the transversal plane, are provided as full 3-D active dose delivery modality. Three rooms with horizontal (and vertical, in one case) fixed beam lines are available for patient treatment. Image-based treatment planning is performed using the commercial Syngo RT Planning system, version VB10 (Siemens AG Healthcare, Erlangen, Germany), supporting three different plan optimization techniques: single field uniform dose (SFUD), patch fields and 3-D intensity modulated particle therapy (IMPT) [18]. Once the TPS and radiation beams were fully commissioned, in September 2011, patient treatments using proton beams started, while the beginning of the clinical activity using carbon ions is very recent (November 2012). So far, about forty adult patients affected by chordoma, chondrosarcoma or squamous cell carcinoma in the head and neck or spine region have been treated.

To avoid adding further complexity, in this analysis, the process tree was defined only considering disease sites not affected by significant organ motion and in adult patients, not needing anaesthesia. In particular, organ motion management and related mitigation techniques for dynamically scanned particle beams, due to the strong interference effect, are still debated and their routine application in the clinical practice is probably premature [19].

Moreover, as a pre-requirement for this analysis, the TPS commissioning was assumed as successfully performed.

## Results and discussion

**Figure 1:**
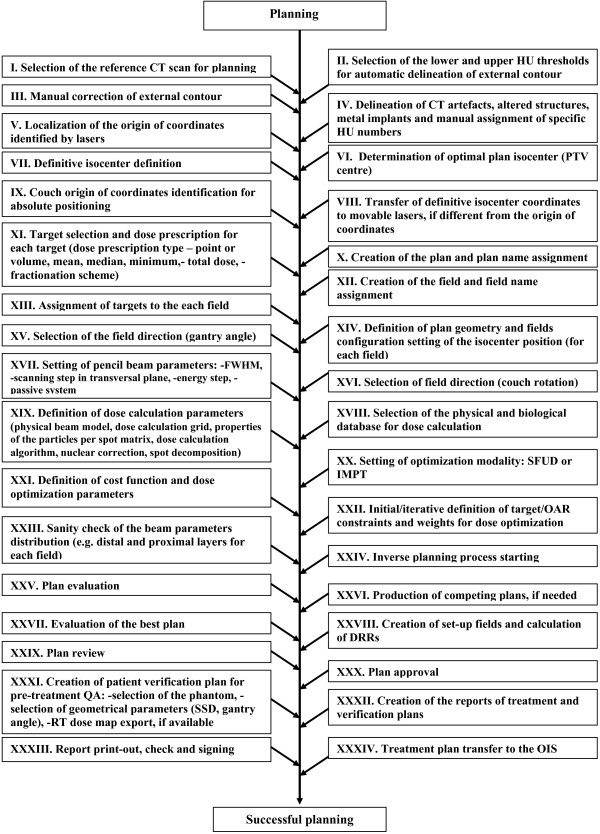
Sub-processes of the treatment planning stage in scanned proton beam radiotherapy.

The process tree of the treatment planning stage is shown in Figure 1. Thirty-four sub-processes were identified, starting from the selection of the reference CT scan for planning, up to the transfer of the approved treatment plan to the Oncology Information System (OIS). Twenty-two of these sub-processes (65% of cases) were judged to be potentially prone to one or more failure modes. The O, S and D indexes for each failure mode were assigned by taking into account the current specific functionalities of Syngo RT TPS as well as the following ten main strategies applied in the clinical practice at CNAO for risk mitigation: (a) definition of detailed site-specific planning protocols and check lists, (b) refusal of enrolling patients with metallic implants disabling accurate delineation of volumes of interest and/or creating unacceptable artefacts in planning CT data, (c) independent double-checking of the main planning parameters used for each individual patient, (d) treatment plan review by the radiation oncology and the medical physics staff before plan approval, (e) skill-based qualitative evaluation of plan robustness, (f) patient treatment position reaching in automatic mode, (g) daily patient set-up verification in the treatment room, (h) isocenter marking on patient’s thermoplastic mask and visual check using lasers (CT-simulation and treatment rooms), (i) regular and successful performing of Department QA checks, (j) clinical utilization of TPS restricted to qualified planners (dosimetrists and medical physicists) who have been trained on its use and limitations.

Following the independent assignment of O, S, and D values for the identified failure modes by each member of the working group, global consensus was easily reached, although a full-day plenary session was needed. Each index was discussed in detail until definitive score achievement, starting from the average of the individual values previously assigned. More than trying to establish universally reliable and objective assignments of the indexes, the attention of the members was paid to reach a proper evaluation consistency within all the failure modes. As already pointed out by the Task Group 100 of the AAPM [14], the RPN consensus values here reported should not be regarded as directly applicable to other centres, unless carefully reviewed by taking into account local specificities.

Globally, forty-four failure modes were identified. In twenty-one cases (48%), quite a low RPN value (range: 18–75) was estimated, so these failures were considered of little concern and reported in Table 1 in a condensed form: they included, for example, the lack of removal of metallic markers from body contour and incorrect localization of the origin of coordinates identified by lasers. Among the potential causes of failure for such minor events, we respectively recognized human error, lack of communication and inadequate operator skill, while their effects consisted of wrong dose distribution (i.e. deviating from the planned one) delivered to the patient, unintended normal tissue irradiation, target geographical missing or underdose, low plan robustness and sub-optimal plan quality.

**Table 1:** Application of failure mode and effects analysis for the treatment planning stage in proton beam radiotherapy

**Sub-process**	**Potential failure mode**
(III) Manual correction of external contour	Lack of removal of metallic skin markers
(V) Localization of the origin of coordinates identified by lasers	Incorrect localization of the origin of coordinates identified by lasers (small amount, 1–2 mm)
	Incorrect localization of the origin of coordinates identified by lasers (large amount)
(VIII) Transfer of definitive isocenter coordinates to movable lasers if different from the origin of coordinates	Lack of information transfer (no virtual simulation)
	Wrong data transfer
	Overwrite of file data
(XI) Target selection and dose prescription setting for each target (dose prescription type - point or volume, mean, median, minimum, - total dose, - fractionation scheme)	Incomplete target selection
	Wrong target selection
	Wrong target dose prescription
(XIII) Assignment of targets to the field	Incomplete or wrong target assignment
(XIV) Field isocenter position setting	Incorrect isocenter definition
(XV - XVI) Selection of field direction (gantry angle and couch rotation)	Improper selection of gantry angle/couch rotation: tissue interfaces lying parallel to beam direction, when otherwise avoidable (i.e. when more robust alternative geometry would be feasible)
	Improper selection of gantry angle/couch rotation: not reachable position (potential collision or movement limitations)
(XVII) Setting of pencil beam parameters:	
- Full Width Half Maximum (FWHM)	Improper selection of beam parameters: FWHM, scanning or energy step too low in relationship to the PTV volume
- scanning step in the transversal plane	
- energy step	
- passive elements (range shifter and ripple filter)	
(XVIII) Selection of the physical and biological database for dose calculation	Selection of unapproved (i.e. not validated for clinical practice, for experimental use only) database.
(XX) Setting of the optimization modality (SFUD, IMPT)	Improper setting of the cost function parameters
	Improper selection of optimization modality rather than IMPT
(XXVII) Evaluation of the best plan	Improper selection of the best plan among the competing calculated onces in terms of optimal trade-off between plan quality (PTV dose coverage *versus* OAR sparing) and robustness
(XXVIII) Creation of set-up fields and calculation of DRRs	Wrong definition of field isocenter (large amount)
	Improper selection of parameters for DRR calculation
(XXXI) Selection of the phantom for the verification plan	Improper phantom selection

Failure modes having an assigned RPN value lower than 80 are listed.

Twenty-three failures modes (52% of cases) were characterized by an RPN score equal to 80 or higher, as shown in Table 2. The mean values of O, S and D parameters were 3.6, 6.9 and 4.7, respectively, suggesting that such events, on average, are infrequent (once or few times a year) and not too difficult to detect, but potentially severe in terms of patient safety. The threshold of 125 for RPN was exceeded in five cases only. The highest RPN values, equal to 192 and 196, were associated to an outdated representation of patient anatomy and incorrect proton stopping power assignment to body regions, due to artefacts, altered structures, or metal implants within the planning CT scan, respectively. Both failure modes appeared moderately infrequent, potentially severe and difficult to detect.

**Table 2:** Application of failure mode and effects analysis for the treatment planning stage in proton beam radiotherapy

**Sub-process**	**N**	**Potential failure mode**	**Potential causes of failure**	**Potential effects of failure**	**O**	**S**	**D**	**RPN**
(I) Selection of the reference CT scan for planning	1	Error in selecting the CT scan (e.g. incorrect patient set up, outdated representation of the anatomy) in case of multiple CT scans	Human error, failure in the communication between operators	Wrong dose distribution/wrong dose delivery	**3**	**8**	**4**	**96**
	2	Outdated representation of the anatomy (single CT scan)	Anatomical changes (related to time delay)	Wrong dose distribution/wrong dose delivery	**3**	**8**	**8**	**192**
(III) Manual correction of external contour	3	Incorrect external contour definition (body or patient mask countour underestimation, i.e. not fully included in the external contour)	Human error	Wrong dose distribution / wrong dose delivery	**4**	**5**	**4**	**80**
	4	Failure of object/region identification	Human error	Wrong dose distribution	**3**	**8**	**4**	**96**
	5	Inaccurate delineation	Human error	Wrong dose distribution	**4**	**6**	**6**	**144**
(IV) Delineation of CT artefacts, altered structures, metal implants and manual assignment of specific HU numbers								
	6	Incorrect HU number manual assignment	Human error or lack of documentation from the referring clinicians (e.g. surgeons)	Wrong dose distribution	**4**	**7**	**7**	**196**
	7	Lack of couch origin of coordinates definition	Human error	Unintended normal tissue irradiated and CTV missing	**3**	**10**	**3**	**90**
(IX) Couch origin of coordinates identification for absolute positioning	8	Wrong definition of couch origin of coordinates (large amount)	Human error	Unintended normal tissue irradiated and CTV missing	**3**	**10**	**3**	**90**
	9	Wrong definition of couch origin of coordinates (small amount, in terms of 2–3 mm)	Human error	Unintended normal tissue irradiated and CTV missing	**4**	**5**	**6**	**120**
(XI) Target selection and dose prescription for each target (dose prescription type - point or volume, mean, median, minimum-, total dose, fractionation scheme)	10	Wrong setting of dose prescription type	Human error	Wrong dose delivery	**3**	**8**	**4**	**96**
	11	Wrong dose fractionation setting	Human error and lack of verbal-written communication (patient chart)	Wrong dose delivery	**2**	**10**	**4**	**80**
	12	Improper selection of gantry angle/couch rotation: beam passing through OARs, when otherwise avoidable	Inadequate operator skill	Sub-optimal treatment quality: increased treatment toxicity	**4**	**6**	**4**	**96**
(XV - XVI) Selection of field direction (gantry angle and couch rotation)	13	Improper selection of gantry angle/couch rotation: beam stopping against OARs, when otherwise avoidable	Inadequate operator skill	Low plan robustness (range uncertainty)	**4**	**8**	**4**	**128**
	14	Improper selection of gantry angle/couch rotation: beam passing through unstable tissues (such as bowel), when otherwise avoidable	Inadequate operator skill	Low plan robustness (range uncertainty)	**3**	**8**	**5**	**120**
(XVII) Setting of pencil beam parameters:	15	Improper selection of beam parameters: FWHM, scanning or energy step too large in relationship to PTV volume	Inadequate operator skill	Sub-optimal treatment quality: increased treatment toxicity or reduced TCP	**4**	**5**	**4**	**80**
- FWHM								
- scanning step in the transversal plane								
- energy step								
- passive elements								
(XIX) Definition of dose calculation parameters:	16	Improper selection of physical beam model and/or calculation grid	Human error due to time pressure or inadequate skills	Wrong dose distribution	**4**	**7**	**5**	**140**
- physical beam model	17	Improper selection of properties of the particles per spot matrix	Human error	Sub-optimal treatment quality	**4**	**4**	**5**	**80**
- dose calculation grid								
- properties of the particles per spot matrix								
- dose calculation algorithm, nuclear correction, spot decomposition								
(XX) Setting of the optimization modality (SFUD, IMPT)	18	Improper selection of IMPT modality	Inadequate operator skill	Low plan robustness: increased treatment toxicity or reduced TCP	**4**	**5**	**4**	**80**
(XXII) Initial/iterative definition of target/OAR constraints and weights for dose optimization	19	Wrong or incomplete definition of one or more dose constraints	Inadequate operator skill or inattention	Sub-optimal treatment quality	**4**	**8**	**3**	**96**
(XXV) Plan evaluation	20	Improper acceptance of results	Time pressure or inadequate operator skill	Sub-optimal treatment quality	**5**	**4**	**4**	**80**
(XXVI) Production of competing plan	21	Lack of producing enough competing plans	Time pressure or inadequate operator skill	Sub-optimal treatment quality	**5**	**4**	**4**	**80**
(XXVIII) Creation of set-up fields and calculation of DRRs	22	Wrong definition of field isocenter (small amount 2 mm)	Human error	Wrong dose delivery	**3**	**5**	**8**	**120**
(XXX) Plan approval	23	Approval of wrong plan	Human error, failure in the communication between operators	Wrong delivery	**3**	**8**	**5**	**120**

Failure modes having an assigned RPN ≥80 are reported.

Three cases (failures n. 7, 8 and 11) could lead to very severe injuries or even patient death under worst conditions (delivered biological dose approximately deviating by more than 15% or 10 GyE from the total prescribed one) and consisted of lack or wrong localization of couch origin of coordinates by a large amount (more than 3 mm, corresponding to the typical organ-at-risk safety margin) and wrong dose fractionation scheme setting. On the other side, the estimated overall RPN for those failure modes did not exceeded the value of 90, since they appeared easily detectable by means of plan review, in-room patient set-up verification procedures and pre-treatment patient-specific dosimetric QA checks.

The most critical sub-process within the treatment planning stage appeared related to the delineation and correction of artefacts in planning CT data (n. IV in Table 2): the high RPN values estimated for the three failures associated to that sub-process reflect the very strong sensitivity of particle beams to range uncertainties, unlike in conventional photon RT [20-22]. Potential causes of these failures are represented by human error made by the planner and lack of exhaustive clinical documentation about the previous surgical procedure. Two additional strategies were suggested by the working group for risk mitigation. The first one simply consisted of systematically asking preliminary information to each patient and collecting documentation about any known implanted prosthesis. Secondly, to investigate the utilization of dedicated image reconstruction algorithms on CT/MRI scanners or megavoltage CT imaging to decrease the effects of artefacts and allow more accurate delineation, associated to appropriate material over-writing, of the artefacts themselves and metal implants.

As a further priority, additional safety measures were also investigated to mitigate the risk for the remaining three failure modes (n. 2, 13 and 16) characterized by an RPN higher than 125. Concerning the outdated representation of patient anatomy, due to changes occurring in the period between CT scan and treatment start, the proposed solution simply consisted of enforcing a short interval time (i.e. not more than 7–10 days) between the two phases and, in doubtful cases, plan recalculation on a new CT scan acquired just a few days before the first treatment session. This latter strategy could at least half the D index and consequently the RPN itself.

For the failure mode consisting of improper selection of beam direction, leading to an otherwise avoidable situation in which the spread-out-Bragg-peak (SOBP) stops against one or more organs at risk, the only additional strategy appearing able to significantly reduce the risk consisted of quantitative evaluation of treatment plan robustness, that is the determination of the degree of sensitivity of the plan to the uncertainties involved in the treatment process. They mainly include range, patient set-up, dose calculation and delivery deviations. Nonetheless, that solution did not seem easily and immediately applicable: although several methods (such as minimax, worst case or multi-criteria optimization, probabilistic treatment planning) have been recently proposed for handling the uncertainties in proton therapy [21-26], the inclusion of robustness in the plan optimization process does not yet represent the state-of-art of commercial TPSs for protons. Therefore, additional human and technological resources have probably to be provided to implement those techniques in the clinical practice. In parallel, the role of Monte Carlo simulations is strongly increasing as a support to TPS analytical dose calculation engines [23,27]: Monte Carlo approach seems to represent a flexible modality to analyse plan robustness, by simulating several combinations of uncertainties. In this case too, efforts and time are required to achieve the needed expertise, but at least more complex plans will benefit of it, in terms of reduced sensitivity to the uncertainties, including those due to biological effects (i.e. the increase in the RBE at the distal part of the spread-out-Bragg-peak, while TPSs typically assume a fixed RBE value equal to 1.1) [23].

Finally, the failure mode related to the improper selection of physical beam model or dose calculation grid was recognized as the result of a human error, on its turn due to time pressure or inadequate skill of the planner. The implementation of independent methods for dose calculation, such as again Monte Carlo simulations [23,27], as well as procedures aiming at plan accuracy verification under realistic conditions, as recently proposed by Albertini *et al*. [20], in our opinion could represent additional strategies to increase both the probability of failure detection and the levels of attention and awareness of the planners.

## Conclusions

The application of FMEA to the treatment planning stage in scanned proton beam RT lead to the identification and deep investigation of several failure modes; the assignment of a score assessing the potential risk for each event allowed to rank these failure modes in order of importance and define priorities for risk mitigation with the aim to optimize quality management workflow. In addition to the safety strategies already adopted in the clinical practice and reported in this work, novel solutions have been proposed to increase patient safety. The multi-disciplinary and multi-institutional approach followed in this study appeared quite useful as a mutual experience exchange in a relatively new and complex field, such as actively scanned particle beam RT.

This study was carried out considering the specific processes implemented at CNAO, therefore, the detailed definition of failure modes and the assignment of RPN scores, strongly depend on the specific process under investigation and on the current strategies/solutions locally applied. However, the process and fault trees here delineated can be easily adapted by other users to their local scenario or, at least, be useful as a starting reference point, thus minimizing the workload impact of the FMEA analysis on the involved team.

## Abbreviations

CT: Computed tomography; HU: Hounsfield units; CTV: Clinical target volume; DRR: Digitally reconstructed radiograph; FWHM: Full-width-half-maximum; IMPT: Intensity modulated particle therapy; OAR: Organs at risk; OIS: Oncology information system; PTV: Planning target volume; QA: Quality assurance; RPN: Risk probability number; RT: Radiation therapy; SFUD: Single field uniform dose; SSD: Source-surface-distance; TCP: Tumour control probability.

## Competing interests

The authors declare that they have no competing interests.

## Authors’ contributions

All authors contributed to the FMEA analysis through the definition of the process tree, failure mode identifications and scoring, and suggesting the additional safety measures. MCC coordinated the working group. MC and IV drafted the manuscript. All authors read and approved the final manuscript.
